# Adrenal insufficiency in acute oral opiate therapy

**DOI:** 10.1530/EDM-13-0071

**Published:** 2014-01-01

**Authors:** Caterina Policola, Victoria Stokes, Niki Karavitaki, Ashley Grossman

**Affiliations:** Department of Endocrinology and Metabolic DiseasesUniversità Cattolica del Sacro CuoreRomeUK; 1 Oxford Centre for Diabetes, Endocrinology and MetabolismOxford University Hospitals NHS TrustOld Road, Oxford, OX3 7LEUK

## Abstract

Opiate drugs such as morphine are in extensive use for pain relief and palliation. It is well established that these drugs can cause changes in endocrine function, but such effects are not always sufficiently appreciated in clinical practice, especially in relation to the hypothalamic–pituitary–adrenal (HPA) axis. Herein, we report on an 18-year-old man who was diagnosed with a slipped left femoral epiphysis following a long history of pain in his leg. On examination, he was thought to look relatively young for his age and therefore the orthopaedic surgeons arranged an endocrine assessment, which showed an undetectable concentration of serum cortisol and a suppressed concentration of testosterone; therefore, he was referred urgently with a diagnosis of hypopituitarism. We elicited a history that he had been treated with opiate analgesics for 3 days at the time of his original blood tests. Full endocrine assessment including a short Synacthen test revealed that he now had normal adrenal and pituitary function. We conclude that his morphine therapy had caused profound suppression of his HPA and pituitary–gonadal axes and suggest that clinicians should be aware of these significant changes in patients on even short-term opiate therapy.

**Learning points:**

- Therapy with opiates is the standard therapy for severe acute and chronic pain.
- Such drugs cause profound changes in endocrine function.
- Importantly, opiates suppress the HPA axis at a central level.
- Short-term therapy with morphine could be the cause of biochemical adrenocortical insufficiency.
- Morphine and related drugs also suppress the pituitary–gonadal axis.
- After discontinuation of therapy with such drugs, adrenal function improves.

## Background

Opiates are important agents in the treatment of pain and have also been used for centuries as substances of abuse (1). They play an important role in the management of chronic and acute pain in many patients, including those with cancer in all age groups (2). Opiate-induced effects in the endocrine system are common and have been recognised for more than a century, but are still poorly understood; these effects represent one of the several described side-effects and possible toxicities associated with the chronic use of opiates (3). Although their effects on gonadal function are well recognised, herein, we present a case of profound adrenal failure after short-term therapy with morphine.

## Case presentation

An 18-year-old man with a suspected diagnosis of hypopituitarism was referred by the orthopaedic team. He had initially presented to the Emergency Department with acute-on-chronic pain in his leg following a fall from his bicycle. He had taken codeine 30 mg four times daily for 1 day before presenting to the hospital. He was given 30 mg codeine at 1400 h, 10 mg oral morphine at 1450 h and 15 mg oral morphine at 1555 h that day. He was diagnosed with a slipped upper femoral epiphysis and operated on by the orthopaedic team. On the day of his operation, he was given 3 mg i.v. morphine immediately post-operatively and then 60 mg oral morphine and 100 mg tramadol in divided doses throughout the day. On the following day, he continued to experience pain and was given 50 mg oral morphine and 100 mg tramadol throughout the day. On day 3 post-operatively, suspicions were raised as he appeared young for his age and was relatively old to present with a slipped upper femoral epiphysis. His basal pituitary function was assessed and found to be abnormal. He was started on hydrocortisone therapy and discharged home with a small supply of tramadol 100 mg on an ‘as-required’ basis, with endocrine follow-up being arranged. Endocrine follow-up took place 1 month later. On examination at endocrine follow-up, he looked healthy and had good muscular development, but appeared to be a little young for his age, had rather sparse body hair, well-developed pubic and genital hair, and a normal penis and testicles, which were bilaterally 15 ml. He reported a gradual increase in body bulk and hair, regular morning erections and occasional shaving; his voice changed about the same age as his peers, and he had normal eyesight and no headaches. He did not have any other significant past medical, family or social history, and the rest of the examination was unremarkable.

## Investigation

The orthopaedic surgeons found extremely low concentrations of cortisol and testosterone (no inhalers or creams). His serum cortisol concentration was 15 nmol/l (normal range: 180–620), testosterone concentration <0.4 nmol/l (normal range: 8.4–28.7), luteinizing hormone (LH) concentration 1.1 IU/l (normal range: 1.5–9.3), follicle-stimulating hormone (FSH) concentration 2.2 IU/l (normal range: 2.0–20), thyroid-stimulating hormone (TSH) concentration 0.56 mU/l (normal range: 0.35–5.5), free thyroxine concentration 13.7 pmol/l (normal range: 11–19.5), free tri-iodothyronine concentration 2.7 pmol/l (normal range: 3.5–6.5), insulin-like growth factor 1 concentration 48.2 nmol/l (normal range: 13.6–64.1), and prolactin concentration 169 mU/l (normal range: 45–375). He was started on 10 mg hydrocortisone in the morning, 5 mg at lunchtime and 5 mg in the evening. After 3 months, he was readmitted for further treatment of his hip. He had discontinued taking opiate analgesics at least 2 months earlier and was taking only hydrocortisone and paracetamol. Pituitary function was re-checked before he took his morning dose of hydrocortisone. He underwent a short Synacthen test: this revealed a baseline cortisol concentration of 293 nmol/l, which increased to 589 nmol/l at 30 min and to 702 nmol/l at 60 min. His testosterone concentration was 12.2 nmol/l, LH concentration 4.1 IU/l, FSH concentration 4.4 IU/l, and TSH concentration 2.54 mIU/l. He had a pituitary magnetic resonance imaging scan that revealed a normal pituitary gland.

## Treatment

Hydrocortisone treatment was discontinued and analgesics were no longer required.

## Outcome and follow-up

He was reassured that he was not hypopituitary, and he remains well in the absence of replacement therapy.

## Discussion

Our case report suggests that short-term therapy with morphine may mimic adrenocortical failure. It is known that opioids and opiates inhibit the hypothalamic–pituitary–adrenal (HPA) axis at multiple levels. The reduced concentrations of CRH and vasopressin reduce the secretion of adrenocorticotropic hormone (ACTH) from the pituitary and the capacity of the pituitary to respond to stimulation and directly interfere with adrenal gland production of cortisol and DHEA independently or by CNS down-regulation. Evidence in the literature also suggests that opiates may interfere with the release of adrenal androgens such as DHEAS (3). According to Tsagarakis *et al*. (4), the predominant effect of morphine on hypothalamic CRH-41 release *in vitro* is the suppression of the release induced by a variety of putative neurotransmitters and depolarising agents. Opioid-induced inhibition of the HPA axis is mediated by different mechanisms at the hypothalamic level, with evidence being in favour of δ- and κ-opiate receptor involvement in the control of ACTH release (5), and Delitala *et al*. (6) observed a drop in serum cortisol concentrations following therapy with morphine and other opiates in humans. Recently, Debono *et al*. (7) have reported a case of a 21-year-old female with tramadone-induced adrenal insufficiency. Another case report has described a patient with back pain on fentanyl application who presented adrenocortical insufficiency (8). A similar observation has been made in a patient on hydromorphone therapy (9).

We conclude that the use of potent opiate analgesics, even in the short term, may profoundly suppress the HPA axis as well as the pituitary–gonadal axis, and this may rapidly reverse on cessation of therapy. Clinicians need to be aware of these potent effects if they are to avoid the fallacious diagnosis of hypopituitarism.

## Patient consent

The authors confirm that written informed consent was obtained from the patient for publication of the submitted article and accompanying images through his signature on their consent form.

## Author contribution statement

Dr C Policola attended on the patient and wrote the first draft; Dr V Stokes analysed all patient data; Dr N Karavitaki and Prof. A Grossman supervised the care of the patient.
